# Artificial intelligence in emergency department triage: a scoping review on workload reduction and patient safety enhancement

**DOI:** 10.7586/jkbns.25.045

**Published:** 2025-08-27

**Authors:** Seoyoung Kim, Soo-Hyun Nam, Jungmin Lee

**Affiliations:** 1 Department of Artificial Intelligence Convergence, Graduate School, Hallym University, Chuncheon, Korea; 2 School of Nursing Science, Gyeongkuk National University, Andong, Korea; 3 School of Nursing, Hallym University, Chuncheon, Korea

**Keywords:** Artificial intelligence, Emergency service, hospital, Triage, Patient safety, Workload

## Abstract

**Purpose:**

This scoping review aimed to evaluate current evidence regarding the application of artificial intelligence (AI)-based triage systems in emergency departments (EDs), with a focus on their contributions to workload reduction, patient safety, and decision-making accuracy from a nursing perspective.

**Methods:**

A scoping review was conducted in accordance with PRISMA-ScR guidelines. Six electronic databases (PubMed, CINAHL Plus with Full Text, JSTOR, IEEE Xplore, ProQuest, and Web of Science) were searched for articles published between January 2014 and December 2024. Studies were included if they applied AI techniques to ED triage and reported outcomes related to workload, safety, or triage performance. Data were extracted and thematically analyzed to identify key contributions of AI-based triage systems. Eight studies met the inclusion criteria.

**Results:**

Three major themes were identified: (1) improvement in decision-making accuracy through AI-assisted triage models, (2) reduction in clinician workload, and (3) enhanced identification of critically ill patients contributing to patient safety. Some models achieved high predictive performance, with Area Under the Receiver Operating Characteristic Curve scores reaching up to 0.96. However, heterogeneity in study designs and limited nurse involvement restricted the generalizability and clinical applicability of these studies.

**Conclusion:**

This review synthesizes existing literature on AI-supported triage systems in emergency care and provides foundational insights into their potential to support nursing decision-making. Future research should focus on nurse-centered system design, usability testing in real-world settings, and evaluation of clinical outcomes to ensure effective and ethical integration into nursing practice.

## INTRODUCTION

The growing demand for efficient patient management in emergency departments (EDs) has highlighted the critical role of triage systems in determining patient priority and ensuring timely care delivery [1]. Proficient and accurate triage performed by ED nurses plays a vital role in enhancing patient safety [2]. Suamchaiyaphum et al. [1] emphasized that the accuracy of triage performed by emergency nurses is essential for prioritizing patient care and providing timely and appropriate treatment. However, traditional triage systems, which rely heavily on the clinical expertise of triage nurses and manual decision-making, often face challenges such as individual differences in decision-making, significant workload burden, and human error [3]. These challenges can delay patient treatment and compromise patient safety, particularly in high-pressure ED environments where timely decisions are vital for positive patient outcomes. Consequently, there is an increasing need for innovative solutions to enhance the precision, efficiency, and reliability of triage processes in EDs.

Artificial intelligence (AI) has emerged as a transformative technology with the potential to address these limitations [4,5]. By leveraging machine learning (ML) and deep learning (DL) algorithms, AI can analyze complex datasets, including electronic health records (EHRs), patient demographics, and clinical parameters, to support evidence-based decision-making. AI-based automated triage systems promise to reduce workload by assisting or replacing manual assessments, improve consistency in patient prioritization, and minimize decision errors that can arise from variability in human judgment [6]. These systems can also offer predictive analytics to identify high-risk patients, enabling healthcare providers to allocate resources more effectively and improve patient safety [7]. For example, clinical decision support systems integrate technological capabilities and medical expertise to enhance decision-making processes, providing actionable insights at the point of care to improve diagnostic accuracy and treatment outcomes [5].

Despite its potential, the integration of AI into ED triage systems faces several challenges, including data quality issues, ethical concerns, and the need for clinical validation of AI models. Understanding the current applications, limitations, and opportunities of AI in this domain is crucial for bridging the gap between traditional triage systems and AI-enhanced solutions.

This systematic review aims to explore the role of AI in automated triage systems within EDs. By synthesizing existing research, the review seeks to evaluate how AI contributes to reducing workload, minimizing decision errors, and improving patient safety. Furthermore, it aims to identify research gaps and propose directions for future studies to advance the integration of AI in ED triage processes.

Specifically, this review addresses the following question: How are AI-based triage systems being applied in EDs to reduce clinician workload, enhance patient safety, and improve decision-making accuracy? As a scoping review, this study aimed to map the existing evidence landscape and identify gaps in the application of AI-based triage systems, rather than to evaluate intervention effectiveness or perform quantitative synthesis.

## METHODS

### 1. Study design

This study was conducted as a scoping review to evaluate the role of AI in automated triage systems within EDs. The review aimed to synthesize existing evidence on AI’s impact on nursing workload reduction, error minimization, and patient safety. The methodology followed the Preferred Reporting Items for Systematic Reviews and Preferred Reporting Items for Systematic Reviews and Meta-Analyses (PRISMA) guidelines [8] to ensure methodological rigor.

### 2. Search strategy

A comprehensive search was conducted across six databases: PubMed, CINAHL Plus with Full Text, JSTOR, IEEE Xplore, ProQuest, and Web of Science. Detailed search strategies for each database are provided in Supplementary Table 1. Studies published between January 2014 and December 2024 in peer-reviewed journals were included. Boolean operators (AND/OR) were used with keywords such as "Artificial Intelligence," "Machine Learning," "Deep Learning," "Triage," and "Emergency Department." Filters for English-language full-text articles were applied. Additional manual searches were conducted on reference lists of key articles to identify studies not properly indexed in databases.

### 3. Eligibility criteria

This scoping review included studies that examined the application of AI in triage systems within EDs. Studies were selected if they focused on AI-driven triage mechanisms designed to reduce healthcare professionals’ workload, minimize clinical errors, or enhance patient safety. Only peer-reviewed full-text articles published in English were considered to ensure the methodological rigor and reliability of the review. Furthermore, studies utilizing structured and unstructured clinical data from real-world emergency settings were prioritized, as they provide a more accurate representation of AI implementation in clinical practice.

### 4. Exclusion criteria

Studies that did not meet the inclusion criteria were systematically excluded. Specifically, research focusing on theoretical AI models without direct clinical application was excluded, as they do not provide insights into real-world triage system implementation. Non-peer-reviewed publications, including opinion pieces, conference abstracts, and editorials, were not considered to maintain the scientific integrity of the review. Additionally, studies conducted on animal models or laboratory simulations without relevance to human emergency triage processes were excluded. Research primarily addressing hospital operational efficiency—such as patient flow management, scheduling optimization, or bed capacity prediction—without a direct impact on triage decision-making was also omitted. Furthermore, studies focusing on AI-driven radiology prioritization rather than direct triage decision-making, as well as those centered on hospital admission predictions or intensive care unit triage without nurse-led AI decision support, were excluded from this review.

### 5. Study selection

The study selection process followed the PRISMA guidelines to ensure methodological transparency and reproducibility. The initial search across six electronic databases—PubMed, CINAHL Plus, JSTOR, IEEE Xplore, ProQuest, and Web of Science—yielded 393 potentially relevant studies. After removing 99 duplicate records, a total of 294 unique articles remained for further evaluation.

In the screening phase, titles and abstracts of the remaining studies were reviewed based on the predefined eligibility criteria. A total of 266 studies were excluded for reasons such as lack of AI application in triage systems, absence of clinical validation, or a primary focus on hospital workflow management rather than direct triage support.

**Figure 1.**
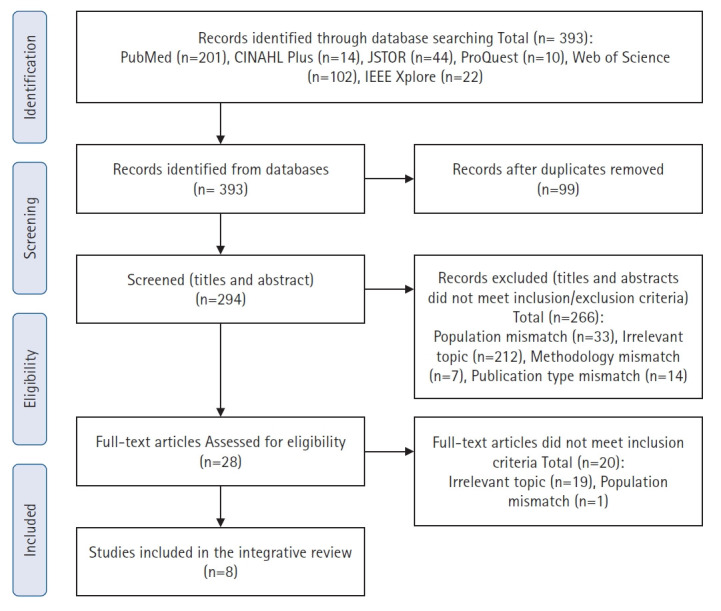
Preferred Reporting Items for Systematic Reviews and Meta-Analyses (PRISMA) flow diagram.

Following this, full-text reviews were conducted on 28 articles, resulting in the exclusion of an additional 20 studies that failed to meet the eligibility criteria. The most common reasons for exclusion at this stage included insufficient data on AI-based triage mechanisms, limited applicability to ED settings, and inadequate reporting of outcome measures related to workload reduction, error minimization, or patient safety. EndNote X20 (Clarivate Analytics, Philadelphia, USA) was utilized for managing citations of the selected articles, and the study selection process is illustrated using a PRISMA flow diagram (Figure 1).

To ensure objectivity and consistency, two independent reviewers performed the study selection process. Any discrepancies in study inclusion decisions were resolved through discussion, with a third reviewer consulted when necessary. This rigorous approach ensured that only the most relevant and methodologically sound studies were included in the final systematic review.

### 6. Risk of bias assessment

To assess the methodological quality and risk of bias within the included studies, the Joanna Briggs Institute Prevalence Critical Appraisal Tool was employed. This tool consists of nine evaluation criteria designed to assess the validity and reliability of prevalence studies. Each study was critically appraised based on the following factors: (1) appropriateness of the sample frame for addressing the target population; (2) adequacy of the sampling method; (3) adequacy of the sample size; (4) detailed description of study subjects and setting; (5) adequacy of data analysis coverage; (6) validity of methods used for condition identification; (7) standardization and reliability of condition measurement; (8) appropriateness of statistical analysis; and (9) adequacy of the response rate and the management of any low response rates. Each criterion was assessed and categorized as "yes," "no," "unclear," or "not applicable," with corresponding justifications provided for each evaluation.

### 7. Data extraction

To ensure consistency and methodological rigor, a structured approach was employed for data extraction. The extraction process aimed to systematically gather relevant information from the included studies, allowing for a comprehensive synthesis of findings. Key study characteristics, including the author(s), year of publication, country, study design, data collection, and comparator, were recorded. In addition, information regarding applied AI technologies, was documented to provide contextual clarity.

Given the focus on AI-driven triage systems, specific details pertaining to the AI models utilized in each study were extracted, including the type of ML algorithm applied, training dataset characteristics, validation methods, and the presence of comparator systems, if applicable. Furthermore, primary and secondary outcome measures were systematically recorded, with particular attention given to workload reduction, error minimization, patient safety improvements, and predictive accuracy metrics.

To enhance the reliability of the data extraction process, two independent reviewers conducted the extraction in parallel. Any discrepancies encountered were resolved through discussion, and when necessary, a third reviewer was consulted to facilitate consensus. Extracted data were systematically organized using Microsoft Excel, ensuring structured and efficient data management for subsequent analysis.

### 8. Data analysis

A comprehensive qualitative data analysis was conducted to synthesize findings from the selected studies and evaluate the impact of AI-driven triage systems in ED settings. The analysis involved the classification of extracted data into key thematic categories, including AI implementation strategies, effectiveness in reducing nursing workload, accuracy in triage decision-making, and overall improvements in patient safety.

## RESULTS

### 1. Overview of AI-based triage systems in EDs

The overall methodological quality of the included studies was deemed acceptable (Table 1). All studies received a “Yes” rating for Q1 (use of an appropriate sample frame) and Q6 (application of valid methods for condition identification). Nonetheless, several studies presented uncertainties in Q2 (sampling strategy) and Q3 (sample size adequacy), with Chen et al. (2023) [9], Chen et al. (2024) [10], and Wu et al. (2021) [11] rated as “Unclear” in one or more of these domains.

Notably, Yu et al. (2020) [4] exhibited the greatest potential for bias, receiving “No” ratings for Q3 and Q5 (completeness of data analysis) and “Unclear” for Q2, Q6, and Q9 (management of response rate), indicating moderate methodological limitations.

These eight studies explored the implementation and impact of AI-based triage systems in ED settings, focusing on outcomes such as reduced ED workload, improved triage decision-making, and enhanced patient safety. Various AI techniques, including DL models, natural language processing, and ensemble ML methods, were employed across different ED settings.

### 2. ED workload reduction

Multiple studies reported that AI-assisted triage can enhance ED workflow efficiency by automating risk stratification and reducing misclassification (Table 2). Chen et al. [10] demonstrated that AI-assisted triage models (Area Under the Receiver Operating Characteristic Curve [AUROC] 0.92) improved patient prioritization, minimized triage errors, and reduced re-triage workload. Similarly, Fernandes et al. [12] found that XGBoost-based triage models outperformed traditional triage methods, achieving higher specificity (0.94 vs. 0.82) and AUROC (0.96 vs. 0.85), suggesting potential workload optimization. Lee et al. [13] developed a ML model (area under the ROC curve = 0.88, balanced accuracy = 0.81) to predict early adverse outcomes in febrile patients, enhancing communication and information sharing among healthcare providers, which facilitated synchronized decision-making and improved operational efficiency. Furthermore, Wu et al. [11] showed that a stacking ML model surpassed the Modified Early Warning Score (MEWS) in predicting in-hospital mortality, with AUROC values of 0.94 at 6 hours and 0.90 at 168 hours, demonstrating superior predictive performance. These findings suggest that AI-based triage systems can help optimize workload distribution and improve overall ED efficiency.

### 3. Patient safety and triage accuracy improvement

AI-based triage systems showed improved performance in identifying critically ill patients compared to traditional scoring methods such as the Emergency Severity Index (ESI) and MEWS. Raita et al. [14] found that ML models outperformed conventional triage approaches, achieving an AUROC of 0.86 for predicting critical care outcomes. Similarly, Chen et al. [10] demonstrated that AI-assisted triage achieved superior AUROC values (0.92) for severe illness prediction compared to human assessments (0.86). Furthermore, subgroup analysis identified patients at relatively higher risk within the same triage level, thereby enhancing patient safety. Additionally, Fernandes et al. [12] reported that XGBoost-based models attained AUROC values up to 0.96, significantly outperforming traditional triage methods. Wu et al. [11] further highlighted that ML models outperformed MEWS in predicting in-hospital mortality across different time points. Yao et al. [15] demonstrated that a DL-based triage system using electronic medical records improved triage accuracy and predictive performance compared to traditional triage methods, particularly in predicting hospital admission (AUROC: 0.87 NHAMCS, 0.88 NTUH), while reducing misclassification errors. These findings suggest that AI-enhanced triage may reduce under-triage errors while improving the accuracy of patient risk stratification.

## DISCUSSION

ED overcrowding is a significant global public health concern, underscoring the importance of triage while revealing the limitations of human-performed triage. This scoping review was conducted to evaluate the role of AI in automated triage systems within EDs. The findings of this scoping review highlight the transformative potential of AI in ED triage systems, particularly in reducing ED workload, improving triage accuracy, and enhancing patient safety. Given the nature of a scoping review, this study did not aim to quantify the efficacy of AI-based triage systems through meta-analysis but rather to explore the breadth of available literature and its clinical implications. By leveraging ML and DL algorithms, AI-based triage systems have demonstrated the capacity to assist healthcare providers in making data-driven decisions, thereby mitigating the limitations associated with traditional, manual triage processes.

One of the key advantages of AI-driven triage is its ability to alleviate the burden on healthcare professionals by automating certain aspects of the decision-making process. In emergent settings, a multi-disciplinary approach is essential, and in the ED, multiple healthcare providers work as a team to treat patients [16]. Several studies included in this review reported that AI-assisted triage systems contributed to a reduction in workload of triage, enabling them to allocate more time to direct patient care [10,12,13]. Since simultaneously treating providers may not always have a clear understanding of each other's intentions, AI-driven triage systems can facilitate information sharing, thereby reducing the workload [13]. By systematically analyzing patient data—such as EHRs, vital signs, and clinical parameters—AI-based triage models have demonstrated improved ability to identify high-risk patients and assist clinical prioritization [12]. Furthermore, these models contribute to standardizing triage assessments, reducing inter-clinician variability and minimizing misclassification risk [17].

Beyond workload reduction, the integration of AI into triage processes has been associated with improvements in patient safety and clinical outcomes. The studies reviewed in this analysis consistently indicated that AI-driven triage systems exhibited superior predictive performance in identifying critically ill patients compared to conventional triage scales such as the ESI and MEWS.

Specifically, AI-based triage systems demonstrated higher sensitivity in identifying patients at risk of severe illness within the same triage level, enabling prioritized treatment and more efficient allocation of medical resources [10]. Additionally, AI-based triage systems can effectively support clinical decision-making, prevent situations where humans may miss critical patients, and ultimately help reduce clinical burden and medical costs [9].

Consequently, the collaborative use of AI-based triage systems and human judgment in ED triage could help mitigate under-triage and over-triage by humans, ultimately enhancing patient safety. From the perspective that over-estimating rather than under-estimating the probability of critical outcomes in high-risk patients is more appropriate for patient prognosis, it was observed that AI-based triage systems tend to over-estimate as the predicted risk increases [12]. By providing real-time risk stratification and predictive analytics, AI algorithms facilitate early recognition of patients who require urgent intervention, potentially preventing delays in treatment [18,19]. The enhanced accuracy of AI-supported triage can contribute to a reduction in under-triage errors, which have been linked to adverse patient outcomes, including delayed access to critical care and increased mortality risk [20].

While the high performance of AI is encouraging, issues of generalizability remain. Compared to prior reviews [21], which often criticized the lack of real-world applicability, our review found that several included studies incorporated actual ED datasets, enhancing ecological validity. Nevertheless, most studies still used retrospective designs, which may introduce biases related to data completeness, accuracy, and generalizability [9]. Moreover, differences in healthcare settings, patient populations, and data collection methods may affect the performance and applicability of AI models across diverse clinical environments [13,14]. Prior studies have also highlighted that while AI-based triage systems show potential to improve efficiency and accuracy in EDs, most research has been limited to single-center studies, with a lack of multicenter validation and standardized outcome reporting [22,23]. To ensure robustness, future research should prioritize the development of AI systems trained on large-scale, multicenter datasets that accurately represent real-world emergency care settings.

Another critical consideration is the interpretability and transparency of AI decision-making processes [13]. Although AI models have demonstrated high predictive accuracy, their "black-box" nature remains a significant barrier to clinical adoption. Healthcare providers must be able to understand and trust AI-generated recommendations before integrating them into patient care workflows. According to previous studies, clinicians’ acceptance of AI-generated recommendations is closely associated with their understanding of the rationale behind those recommendations [24]. In other words, a lack of transparency in AI models may lead to hesitation in trusting and utilizing them in clinical practice, emphasizing that enhancing the interpretability of AI systems is crucial for fostering clinician confidence and facilitating their seamless adoption in clinical environments [25]. Therefore, the incorporation of Explainable AI methodologies could help address this challenge by providing interpretable outputs that justify AI-driven triage decisions [26,27]. Enhancing transparency in AI models is essential for fostering clinician confidence and ensuring that AI recommendations align with established clinical guidelines and best practices.

Ethical and legal implications also warrant careful consideration. The implementation of AI in clinical decision-making raises concerns regarding accountability, particularly in cases where AI-generated recommendations lead to adverse patient outcomes. Questions regarding liability—such as whether responsibility falls on the AI developers, healthcare institutions, or individual clinicians—must be addressed before widespread adoption. Furthermore, issues related to patient privacy and data security must be prioritized, particularly as AI-driven triage systems rely on the integration of large-scale patient data. Regulatory frameworks and standardized guidelines are needed to establish best practices for AI deployment in emergency care settings, ensuring that ethical considerations are adequately addressed.

Future research should focus on the real-world validation of AI-assisted triage systems through prospective clinical trials and implementation studies. While the reviewed studies demonstrate promising predictive accuracy, most were retrospective and conducted in controlled environments. Such conditions cannot fully replicate the dynamic, high-pressure nature of EDs. Therefore, further investigation is needed to evaluate the effectiveness of these systems in live clinical settings. Prospective trials should assess not only predictive performance but also the feasibility, workflow integration, and true impact on patient outcomes. Implementation science approaches are particularly warranted to identify contextual barriers and facilitators to adoption in actual ED environments. The transition from algorithmic performance to clinical utility must be rigorously evaluated before AI-based triage systems can be safely and ethically integrated into nursing workflows.

In addition, hybrid AI-human triage models should be explored to optimize decision-making by combining the strengths of AI-driven analytics with clinical expertise. The integration of AI should not seek to replace human judgment but rather enhance healthcare professionals' ability to make accurate and timely decisions.

This review demonstrates that AI-based triage systems can meaningfully reduce nursing workload and enhance patient safety by improving decision accuracy in emergency care. The integration of AI into triage workflows has the potential to optimize resource allocation, minimize errors, and support timely clinical decisions. From a nursing perspective, these technologies may serve as valuable decision-support tools that complement clinical expertise. The findings highlight the importance of nurse involvement in system development and point to the need for real-world validation to ensure ethical and effective implementation.

## CONCLUSION

This scoping review highlights the expanding role of AI in ED triage, with potential benefits including improved decision-making efficiency, reduced nursing workload, and enhanced patient safety. However, challenges such as data quality, limited transparency of models, and ethical concerns—particularly related to algorithmic bias and clinical accountability—must be addressed to facilitate safe and effective implementation.

From a nursing perspective, AI-based triage systems can serve as decision-support tools that complement, rather than replace, clinical judgment. The findings offer a foundational understanding of how such systems are conceptualized and may contribute to the co-development of nurse-AI collaborative models in emergency settings.

Future research should involve participatory system design with frontline nurses, test usability and acceptability in simulated and clinical environments, and evaluate the impact on workflow integration, autonomy, and patient outcomes. Despite methodological limitations such as heterogeneity in study design and lack of empirical trials, this review provides a critical overview to guide further investigation and innovation in AI-supported nursing triage.

## Figures and Tables

**Table 1.** Risk of Bias Assessment of the Included Studies

Author (year)	Q1	Q2	Q3	Q4	Q5	Q6	Q7	Q8	Q9
Chen et al. (2023) [9]	Y	U	Y	Y	Y	Y	Y	Y	U
Chen et al. (2024) [10]	Y	Y	Y	Y	U	U	U	U	U
Fernandes et al. (2020) [12]	Y	Y	Y	Y	Y	Y	Y	Y	Y
Lee et al. (2020) [13]	Y	Y	Y	Y	U	Y	Y	Y	Y
Raita et al. (2019) [14]	Y	U	Y	Y	Y	Y	Y	Y	Y
Wu et al. (2021) [11]	Y	Y	Y	Y	U	Y	Y	U	Y
Yao et al. (2021) [15]	Y	U	Y	Y	Y	Y	U	Y	U
Yu et al. (2020) [4]	Y	Y	Y	N	Y	Y	Y	Y	Y

Q1 = Was the sample frame appropriate to address the target population?; Q2 = Were study participants sampled in an appropriate way?; Q3 = Was the sample size adequate?; Q4 = Were the study subjects and the setting described in detail?; Q5 = Was the data analysis conducted with sufficient coverage of the identified sample?; Q6 = Were valid methods used for the identification of the condition?; Q7 = Was the condition measured in a standard, reliable way for all participants?; Q8 = Was there appropriate statistical analysis?; Q9 = Was the response rate adequate, and if not, was the low response rate managed appropriately? N = No; U = Unclear; Y = Yes.

**Table 2.** Characteristics of the Included Studies

Author (year); Location	Purpose	Design; data collection	Comparator	Applied AI technologies	Outcome(s)	
					ED workload reduction	Patient safety and triage accuracy
Chen et al. (2023) [9]; Taiwan	Develop a clinical narrative-aware deep neural network to predict critical outcomes in ED patients	Retrospective cohort study; 171,275 adult ED visits from Taipei Medical University-Shuang Ho Hospital (2017~2020)	Emergency physicians’ manual assessment of critical outcomes	Deep neural network (BiLSTM) with NLP (Clinical Narratives Text Representation - CNTR)	AI-assisted triage improved early risk stratification (sensitivity 0.95, accuracy 0.90), reducing physician delays.	AI model achieved AUROC 0.874, surpassing physicians in triage accuracy.
Chen et al. (2024) [10]; Taiwan	Develop a collaborative ML model for ED patient stratification based on severe illness risk	Retrospective study; 668,602 ED visits from a tertiary teaching hospital (2015~2022)	Traditional 5-level TTAS-based triage	Artificial neural network (ANN) models;	ANN-MH model enhanced triage efficiency (AUROC 0.918), reducing misclassification and re-triage workload.	ANN-MH improved AUROC (0.918 vs. 0.857), reducing under-triage.
				ANN-MH (with TTAS), ANN-MO (without TTAS)		
Fernandes et al. (2020) [12]; Portugal	Develop a machine learning model integrating NLP to predict risk of mortality and cardiopulmonary arrest in ED patients	Retrospective cohort study; 235,826 adult ED patients from Hospital Beatriz Ângelo (2012~2016)	Manchester Triage System (MTS) priority-based assessment	Logistic Regression, Random Forest, Extreme Gradient Boosting (XGBoost), NLP using TF-IDF	XGBoost model improved triage prediction (AUROC 0.96 vs. 0.85) and specificity (0.94 vs. 0.82), potentially aiding ED resource allocation.	XGBoost model improved triage accuracy (AUROC 0.96 vs. 0.85), reducing under-triage cases compared to MTS.
Lee et al. (2020) [13]; South Korea	Develop a time-adaptive machine learning model to predict adverse outcomes for febrile ED patients using sparse laboratory data	Retrospective cohort study; 9,491 febrile adult ED patients from a tertiary hospital in Seoul (2017~2019)	Modified Early Warning Score (MEWS)	Random forest, elastic net regression (OSO & OSR models)	OSR model improved early risk prediction (AUROC 0.88), reducing delays in triage decision-making.	OSR model improved AUROC (0.88 vs. 0.80), sensitivity (0.81 vs. 0.70), and BA (0.81 vs. 0.74), reducing under-triage.
Raita et al. (2019) [14]; The U.S.	Develop ML models to predict clinical outcomes in ED triage and compare with ESI	Retrospective cohort study; 135,470 adult ED visits from NHAMCS (2007~2015)	Emergency Severity Index (ESI)	Lasso regression, random forest, gradient boosted decision tree, deep neural Network	ML models improved triage efficiency (AUROC 0.86 for DNN), reducing over-triage and improving net benefit.	ML-assisted triage enhanced AUROC (0.86 vs. 0.74), sensitivity (0.86 vs. 0.50), and specificity.
Wu et al. (2021) [11]; Taiwan	Develop an ML-based model to predict in-hospital mortality for non-traumatic ED patients and compare with MEWS	Retrospective multicenter cohort study; 2,437,326 ED visits from 5 hospitals (2008~2016)	Modified Early Warning Score (MEWS)	Stacking ML model (XGBoost, Random Forest, AdaBoost, Logistic Regression)	ML model outperformed MEWS in predicting in-hospital mortality (AUROC 0.939 at 6h), improving early risk stratification.	ML model maintained superior sensitivity, PPV, and NPV over MEWS (p < 0.001), demonstrating better mortality prediction.
Yao et al. (2021) [15]; Taiwan & The U.S.	Develop a deep learning-based triage system using EMR data to predict clinical outcomes after ED treatment	Retrospective cohort study; 118,602 adult ED patients from NHAMCS (2012~2016) and 745,441 patients from NTUH (2009~2015)	Emergency Severity Index (ESI) & conventional ML models	CNN + RNN with attention mechanism	Deep learning triage system utilized EMR data, improving early risk stratification and potentially assisting in ED resource allocation.	Deep learning model achieved AUROCs of 0.87 (NHAMCS) and 0.88 (NTUH), surpassing LR (0.82) and XGBoost (0.84).
Yu et al. (2020) [4]; South Korea	Develop an ML and initial nursing assessment (INA)-based triage system for predicting adverse clinical outcomes in ED	Retrospective single-center study; 86,304 ED visits from a tertiary academic hospital (2016~2017)	Korea Triage and Acuity Scale (KTAS) & Sequential Organ Failure Assessment (SOFA)	Logistic regression, random forest, deep learning	ML and INA-based triage system improved prediction accuracy (AUROC 0.872-0.876), suggesting potential workflow optimization.	INA model surpassed KTAS (AUROC 0.768) and SOFA (AUROC 0.740), reducing misclassification risk.

ED = Emergency department; AI = Artificial intelligence; BiLSTM = Bidirectional long short-term memory; NLP = Natural language processing; CNTR = Clinical narrative text representation; AUROC = Area Under the Receiver Operating Characteristic Curve; TTAS = Taiwan Triage and Acuity Scale; ANN = Artificial neural network; ANN-MH = Artificial neural network - model hybirid; ANN-MO = Artificial neural network - model only; TF-IDF = Term frequency–inverse document frequency; XGBoost = Extreme gradient boosting; MTS = Manchester Triage System; OSO = Outcome stratification – original; OSR = Outcome stratification – revised; MEWS = Modified Early Warning Score; NHAMCS = National Hospital Ambulatory Medical Care Survey; ESI = Emergency Severity Index; ML = Machine learning; DNN = Deep neural network; PPV = Positive predictive value; NPV = Negative predictive value; NTUH = National Taiwan University Hospital; CNN = Convolutional neural network; RNN = Recurrent neural network; LR = Logistic regression; INA = Initial nursing assessment; KTAS = Korea Triage and Acuity Scale.

## Data Availability

All data analyzed in this study were obtained from previously published peer-reviewed articles. The dataset used for the review, including the list of included studies and extracted data, is available from the corresponding author upon reasonable request.

## Supplementary Material

Supplementary Table 1. Search Strategy for the Included Databases
