# Supplementary material for: Artificial intelligence in emergency department triage: a scoping review on workload reduction and patient safety enhancement
Source: J Korean Biol Nurs Sci. 2025 Aug 27;27(3):333–42. doi: 10.7586/jkbns.25.045 (PMC13268333; doi:10.7586/jkbns.25.045)
Supplement: Supplementary file 1 [file jkbns-25-045-Supplementary-Table-1.pdf]

Supplementary Table 1. Search Strategy for the Included Databases

Database: PubMed (n = 201)

No.	Search Strategy	Results
#1	AI OR "Artificial Intelligence" OR "Machine Learning" OR "Deep Learning"	141,793
#2	Triage OR "Emergency Department" OR "Emergency Room" OR "Patient Prioritization"	20,148
#3	"Workload Reduction" OR "Nursing Workload" OR "Job Burden" OR "Task Optimization" OR "Workload Optimization" OR "Medical Errors" OR "Decision Error" OR "Judgment Error" OR "Human Error" OR "Patient Safety" OR "Clinical Outcomes" OR "Risk Reduction"	317,371
#4	#1 AND #2 AND #3	298
#5	#1 AND #2 AND #3 (Limiters - Published Date: 2014.01.01-2024.12.31; English Language; Free Full Text)	201

Database: CINAHL Plus with Full Text (n = 14)

No.	Search Strategy	Results
#1	(MH "Artificial Intelligence" OR "Machine Learning" OR "Deep Learning")	39,102
#2	(MH "Triage" OR "Emergency Department" OR "Emergency Room" OR "Patient Prioritization")	94,995
#3	("Workload Reduction" OR "Nursing Workload" OR "Task Optimization" OR "Medical Errors" OR "Patient Safety")	107,860
#4	S1 AND S2 AND S3	19
#5	S4 (Limiters - Published Date: 2014.01.01-2024.12.31; English Language; Full Text)	14

Database: JSTOR (n = 44)

No.	Search Strategy	Results
S1	"Artificial Intelligence" OR "Machine Learning"	70,006
S2	"Triage" OR "Emergency Department"	34,529
S3	"Workload Reduction" OR "Patient Safety"	14,277
S4	(S1 AND S2) AND S3	50
S5	(S1 AND S2) AND S3 (Limiters - Published Date: 2014-2024; Language: English; Content Access: Open Access)	44

Database: IEEE Xplore (n = 22)

No.	Search Term	Results
S1	"All Metadata":AI OR "All Metadata":"Artificial Intelligence" OR "All	658,893

	Metadata":"Machine Learning" OR "All Metadata":"Deep Learning"	
S2	"All Metadata":"Triage" OR "All Metadata":"Emergency Department" OR "All Metadata":"Emergency Room" OR "All Metadata":"Patient Prioritization"	1,863
S3	"All Metadata":"Workload Reduction" OR "All Metadata":"Nursing Workload" OR "All Metadata":"Patient Safety" OR "All Metadata":"Task Optimization" OR "All Metadata":"Workload Optimization"	2,339
S4	S1 AND S2 AND S3	179
S5	S4 (Limiters - Published Date: 2014-2024; Open Access Only)	22

Database: ProQuest (n = 10)

No.	Search Strategy	Results
S1	TI("Artificial Intelligence" OR "AI" OR "Machine Learning" OR "Deep Learning")	1,014,981
S2	TI("Triage" OR "Emergency Department" OR "Emergency Room" OR "Patient Prioritization")	56,513
S3	AB("Workload Reduction" OR "Nursing Workload" OR "Job Burden" OR "Task Optimization" OR "Workload Optimization" OR "Medical Errors" OR "Decision Error" OR "Judgment Error" OR "Human Error" OR "Patient Safety" OR "Clinical Outcomes" OR "Risk Reduction")	116,701
S4	S1 AND S2 AND S3	20
S5	S4 (Limiters - Full Text Only; Published Date: 2014.01.01 – 2024.12.31; Language: English)	10

Database: Web of Science (n = 102)

No.	Search Strategy	Results
S1	TS=("Artificial Intelligence" OR "AI" OR "Machine Learning" OR "Deep Learning")	928,429
S2	TS=("Triage" OR "Emergency Department" OR "Emergency Room" OR "Patient Prioritization")	120,302
S3	TS=("Workload Reduction" OR "Nursing Workload" OR "Task Optimization" OR "Medical Errors" OR "Patient Safety" OR "Clinical Outcomes" OR "Risk Reduction")	222,591
S4	S1 AND S2 AND S3	162
S5	S4 (Limiters – Open Access; Published Date: 2014.01.01 – 2024.12.31; Language: English)	102
